# S-Acetyl-Glutathione Attenuates Carbon Tetrachloride-Induced Liver Injury by Modulating Oxidative Imbalance and Inflammation

**DOI:** 10.3390/ijms23084429

**Published:** 2022-04-17

**Authors:** Rosanna Di Paola, Sergio Modafferi, Rosalba Siracusa, Marika Cordaro, Ramona D’Amico, Maria Laura Ontario, Livia Interdonato, Angela Trovato Salinaro, Roberta Fusco, Daniela Impellizzeri, Vittorio Calabrese, Salvatore Cuzzocrea

**Affiliations:** 1 Department of Veterinary Sciences, University of Messina, 98168 Messina, Italy; dipaolar@unime.it; 2 Department of Biomedical and Biotechnological Sciences, University of Catania, 95124 Catania, Italy; sergio.modafferi@studium.unict.it (S.M.); marialaura.ontario@ontariosrl.it (M.L.O.); calabres@unict.it (V.C.); 3 Department of Chemical, Biological, Pharmaceutical and Environmental Sciences, University of Messina, Viale Ferdinando Stagno D’Alcontres 31, 98166 Messina, Italy; rsiracusa@unime.it (R.S.); rdamico@unime.it (R.D.); linterdonato@unime.it (L.I.); dimpellizzeri@unime.it (D.I.); salvator@unime.it (S.C.); 4 Department of Biomedical, Dental and Morphological and Functional Imaging, University of Messina, Via Consolare Valeria, 98125 Messina, Italy; marika.cordaro@unime.it; 5 Department of Clinical and Experimental Medicine, University of Messina, Via Consolare Valeria, 98125 Messina, Italy

**Keywords:** liver fibrosis, antioxidant, inflammation

## Abstract

Liver fibrosis, depending on the stage of the disease, could lead to organ dysfunction and cirrhosis, and no effective treatment is actually available. Emergent proof supports a link between oxidative stress, liver fibrogenesis and mitochondrial dysfunction as molecular bases of the pathology. A valid approach to protect against the disease would be to replenish the endogenous antioxidants; thus, we investigated the protective mechanisms of the S-acetyl-glutathione (SAG), a glutathione (GSH) prodrug. Preliminary in vitro analyses were conducted on primary hepatic cells. SAG pre-treatment significantly protected against cytotoxicity induced by CCl4. Additionally, CCl4 induced a marked increase in AST and ALT levels, whereas SAG significantly reduced these levels, reaching values found in the control group. For the in vivo analyses, mice were administered twice a week with eight consecutive intraperitoneal injections of 1 mL/kg CCl4 (diluted at 1:10 in olive oil) to induce oxidative imbalance and liver inflammation. SAG (30 mg/kg) was administered orally for 8 weeks. SAG significantly restored SOD activity, GSH levels and GPx activity, while it strongly reduced GSSG levels, lipid peroxidation and H_2_O_2_ and ROS levels in the liver. Additionally, CCl4 induced a decrease in anti-oxidants, including Nrf2, HO-1 and NQO-1, which were restored by treatment with SAG. The increased oxidative stress characteristic on liver disfunction causes the impairment of mitophagy and accumulation of dysfunctional and damaged mitochondria. Our results showed the protective effect of SAG administration in restoring mitophagy, as shown by the increased PINK1 and Parkin expressions in livers exposed to CCl4 intoxication. Thus, the SAG administration showed anti-inflammatory effects decreasing pro-inflammatory cytokines TNF-α, IL-6, MCP-1 and IL-1β in both serum and liver, and suppressing the TLR4/NFkB pathway. SAG attenuated reduced fibrosis, collagen deposition, hepatocellular damage and organ dysfunction. In conclusion, our results suggest that SAG administration protects the liver from CCl4 intoxication by restoring the oxidative balance, ameliorating the impairment of mitophagy and leading to reduced inflammation.

## 1. Introduction

Liver fibrosis is a characteristic result of chronic liver injury. It is caused by numerous insults, including toxic damage, alcohol abuse, viral infection and metabolic disorders. Additionally, it is well described that a substantial increase in fibrosis and steatosis frequently leads to fatal cirrhosis and even hepatocellular carcinoma in humans [1]. The high incidence of these hepatopathies places them as one of the most severe diseases. Although the mechanism of some etiologies of liver fibrosis is not fully described, it is clear that reactive oxygen species (ROS) play a key role in the pathogenesis of liver diseases [2]. Increased ROS levels are involved in many pathological conditions and diseases, including cardiovascular disease, diabetes, aging and cancer [3,4,5]. The repeated administration of carbontetrachloride (CCl4) has become one of the most commonly used experimental models for inducing toxin-mediated liver fibrosis [6]. In many aspects, it mimics human chronic disease associated with toxic damage. CCl4 induces severe liver cell damage through the elevation of ROS, resulting in apoptosis, fibrosis and liver injury that contribute to an acute phase reaction characterized by the necrosis of centrilobular hepatocytes, the activation of Kupffer cells and the induction of an inflammatory response [7]. This sequence is associated with the production of several cytokines that cause liver fibrosis [8]. CCl4 is metabolized in the liver by the cytochrome P450 superfamily of monooxygenases (CYP family) to the trichloromethyl radical (CCl*3). Subsequently, this radical reacts with nucleic acids, proteins and lipids, thereby impairing key cellular processes, resulting in an altered lipid metabolism (fatty degeneration and steatosis). The formation of trichloromethylperoxy radicals (CCl_3_OO*) resulting from the oxygenation of CCl_3_* further initiates lipid peroxidation and the destruction of polyunsaturated fatty acids. Consequently, the membrane permeability in all cellular compartments (mitochondria, endoplasmic reticulum and plasma membrane) is lowered and generalized hepatic damage occurs that is characterized by inflammation, fibrosis and cirrhosis. Biological membranes are particularly prone to the ROS injuries. The peroxidation of fatty acids in cellular membranes induces a decrease in membrane fluidity and disruption of membrane function and integrity, which is involved in severe pathological changes [9]. It is clear that the direct diminution of ROS levels and inhibition of the oxidative chain reaction induced by CCl4 administration may be critical for the treatment and prevention of CCl4-induced liver damage [10]. In fact, it has been previously described that CCl4 injection increased pro-inflammatory mediators and decreased the antioxidant proteins during liver injury and fibrosis [6,11]. Many endogenous protective mechanisms have been characterized to limit ROS-induced damage [12]. However, additional protective mechanisms of exogenous antioxidants may be important. Thus, many artificial and natural agents possessing antioxidative effects have been suggested to treat and prevent hepatopathies induced by excessive oxidative stress [13,14]. Therefore, supplementation with anti-oxidants is beneficial for human health. Glutathione (γ-L-glutamyl-L-cysteinylglycine, GSH) is an endogenous tripeptide and liver is one of the tissues with the highest content of it. In particular, it is the principal tissue involved in its biosynthesis [15]. Within the cell, GSH is maintained in its thiol-reduced form by an NADPH-dependent enzyme (glutathione disulfide (GSSG) reductase). An additional amount of GSH is present as GSSG and as glutathione conjugates (GS-R). Keeping optimal GSH:GSSG ratios is fundamental for cell survival, since GSH is one of the most important endogenous antioxidant defense systems, removing lipid- and hydrogen-peroxides [16]. A valid approach to replenish the endogenous GSH is using S-acyl prodrugs such as S-acetyl-glutathione (SAG). It is able to cross the cell membrane and increase intracellular SH groups [17]. Differently from GSH, which enters the cells directly, SAG is more stable in blood plasma and, once entered into cell cytoplasm, is converted by cytoplasmatic thioesterases to GSH. In particular, the acetylation of the sulfur atom avoids the GSH decomposition and simplifies its absorption via the intestinal wall [18]. 

Therefore, in this study, we evaluated the effects of the SAG administration in a mouse model of liver injury, focusing the attention on the CCl4-induced fibrosis, oxidative stress, mitophagy and inflammation. 

## 2. Results

### 2.1. Effect of SAG on Cytotoxicity and Hepatoprotective Activity in Cells: Preliminary In Vitro Data

**Figure 1:**
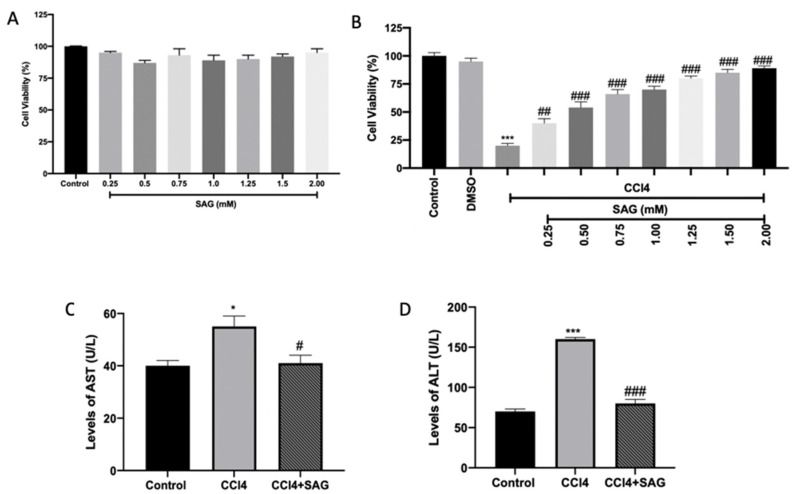
Preliminary data on SAG effect on primary hepatic cells: Viability of cells treated with SAG (**A**); Viability of cells pre-treated for 1 h with SAG followed by exposure to CCl4 (4 mM) for 6 h (**B**); Enzymatic activities of AST (**C**) and ALT (**D**) in the supernatant. The results were analyzed by one-way ANOVA, followed by a Bonferroni post hoc test for multiple comparisons. A *p*-value of less than 0.05 was considered significant. A *p*-value of less than 0.05 was considered significant. ^#^ *p* < 0.05 vs. control, * *p* < 0.05 vs. CCL4, ^##^ *p* < 0.01 vs. control, ^###^ *p* < 0.001 vs. control, *** *p* < 0.001 vs. CCL4.

Primary hepatic cells only exposed to SAG (0.25–2.00 mM) or the CCl4 vehicle (DMSO 0.5%) showed no changes in cell viability (Figure 1A). On the other hand, primary hepatic cells exposed to CCl4 (4 mM) presented a significant reduction in cell viability when compared to the control group. In turn, pre-treatment with all concentrations of SAG followed by exposure to CCl4 significantly protected against cytotoxicity (Figure 1B). 

In primary hepatic cells exposed to CCl4, both AST and ALT levels presented a marked increase, respectively (Figure 1C,D, respectively), in relation to the control group. By contrast, pre-treatment with SAG at 2.00 mM significantly reduced both levels (Figure 1C,D).

### 2.2. Experimental Timeline

**Figure 2:**
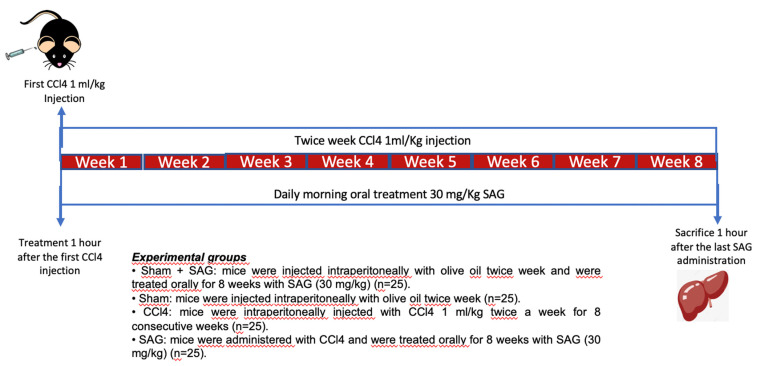
Schematic of study design.

CCl4 is well-known to cause hepatic injury, apoptosis and necrosis [6]. In order to investigate the effects of SAG on hepatic damage, mice were intraperitoneally injected with CCl4 1 mL/kg twice a week for 8 consecutive weeks to induce liver fibrosis and were treated orally for 8 weeks with SAG (30 mg/kg) dissolved in saline (Figure 2). 

### 2.3. Effects of SAG on Oxidative Stress Induced by CCl4 Chronic Exposure

**Figure 3:**
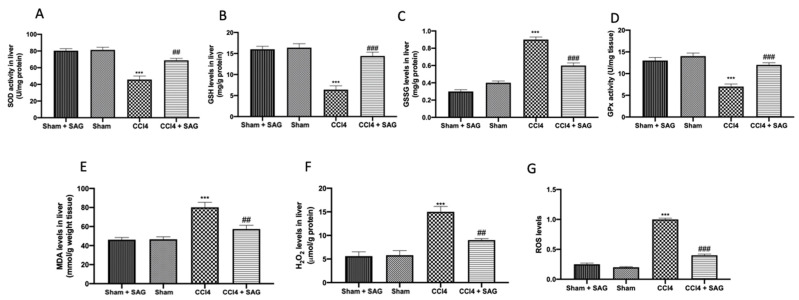
Administration of SAG reduced oxidative stress induced by CCl4 injections: SOD activity (**A**); GSH levels (**B**); GSSG levels (**C**); GPx activity (**D**); MDA levels (**E**); H_2_O_2_ levels (**F**); ROS levels (**G**). For each analysis, *n* = 5 animals for each group were employed. The results were analyzed by one-way ANOVA, followed by a Bonferroni post hoc test for multiple comparisons. A *p*-value of less than 0.05 was considered significant. A *p*-value of less than 0.05 was considered significant. ^##^ *p* < 0.01 vs. sham, ^###^ *p* < 0.001 vs. sham, *** *p* < 0.001 vs. CCL4.

CCl4 intoxication downregulated SOD (Figure 3A) and GSH (Figure 3B) compared to the sham groups. SAG administration significantly restored SOD activity (Figure 3A) and GSH levels (Figure 3B) in the liver. Additionally, CCl4 administration increased GSSG levels, as compared to the sham groups, whereas it was significantly decreased by SAG administration (Figure 3C). Glutathione peroxidase (GPx) activity was impaired after CCl4 chronic exposure, as compared to the sham groups, whereas SAG administration significantly restored it (Figure 3D). On the same line, SAG supplementation reduced lipid peroxidation (Figure 3E), H_2_O_2_ levels (Figure 3F) and ROS levels (Figure 3G) in the samples. 

### 2.4. Effects of SAG on Mitophagy Impairments Induced by CCl4 Chronic Exposure

**Figure 4:**
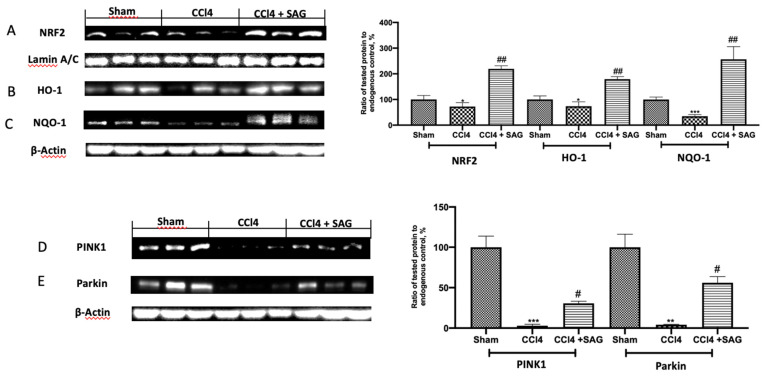
Administration of SAG ameliorated the impaired mitophagy induced by CCl4 injections: Western blot analysis of: NRF2 (**A**), HO-1 (**B**), NQO-1 (**C**), PINK1 (**D**) and Parkin (**E**) levels. For each analysis, *n* = 5 animals for each group were employed. The results were analyzed by one-way ANOVA, followed by a Bonferroni post hoc test for multiple comparisons. A *p*-value of less than 0.05 was considered significant. A *p*-value of less than 0.05 was considered significant. ^#^ *p* < 0.05 vs. sham, * *p* < 0.05 vs. CCL4, ^##^ *p* < 0.01 vs. sham, ** *p* < 0.01 vs. CCL4, *** *p* < 0.001 vs. CCL4.

Western blot analysis displayed that the CCl4 induced a decrease in anti-oxidants, including Nrf2 (Figure 4A), HO-1 (Figure 4B) and NQO-1 (Figure 4C), which were restored by treatment with SAG. Western blot analysis was also employed to evaluate the effect of SAG on the mitochondrial homeostasis. Chronic CCl4 exposure impaired mitophagy and mitochondrial biogenesis. PINK1 (Figure 4D) and Parkin (Figure 4E) levels were decreased following CCl4 intoxication, whereas SAG administration significantly increased their levels. 

### 2.5. Effects of SAG on Pro-Inflammatory Mediator Secretion Induced by CCl4 Chronic Exposure

**Figure 5:**
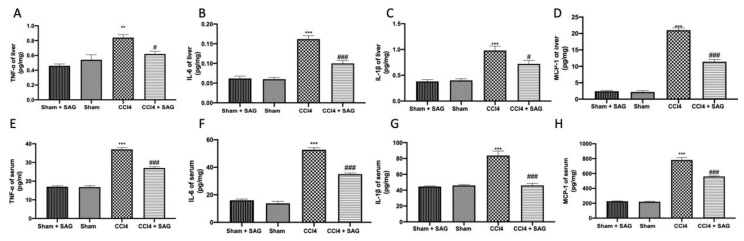
Administration of SAG reduced pro-inflammatory mediator secretion induced by CCl4 injections: Liver levels of TNF-α (**A**), IL-6 (**B**), IL-1β (**C**) and MCP-1 (**D**); Serum levels of TNF-α (**E**), IL-6 (**F**), IL-1β (**G**) and MCP-1 (**H**). For each analysis, *n* = 5 animals for each group were employed. The results were analyzed by one-way ANOVA, followed by a Bonferroni post hoc test for multiple comparisons. A *p*-value of less than 0.05 was considered significant. A *p*-value of less than 0.05 was considered significant. ^#^ *p* < 0.05 vs. sham, ** *p* < 0.01 vs. CCl4, ^###^ *p* < 0.001 vs. sham, *** *p* < 0.001 vs. CCl4.

ELISA analysis showed elevated levels of TNF-α (Figure 5A), IL-6 (Figure 5B), IL-1β (Figure 5C) and MCP-1 (Figure 5D) in the liver after CCl4 intoxication, which were reduced by SAG administration. In addition, the circulating pro-inflammatory cytokines (Figure 5E, 5F and 5G, respectively), as well as the chemokine (Figure 5H), were significantly increased in the CCl4-treated mice compared to the sham group. However, mice treated with SAG exhibited a significant downregulation of pro-inflammatory mediators in the serum. 

### 2.6. Effects of SAG on TL4/NFkB Signaling Activation Induced by CCl4 Chronic Exposure

**Figure 6:**
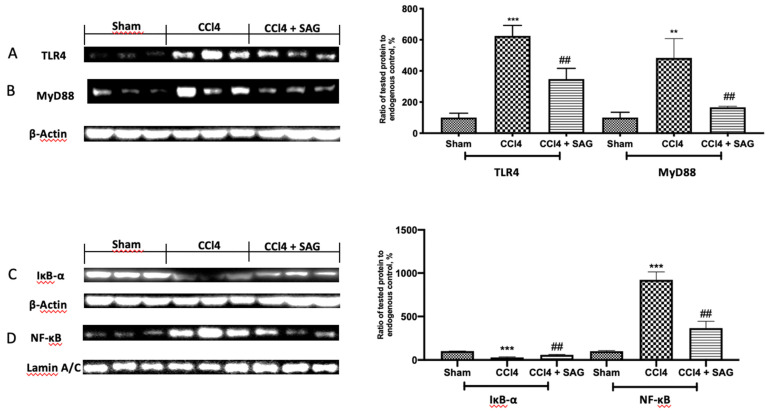
Administration of SAG reduced the activation of the TLR4/NF-kB pathway induced by CCl4 injections: Western blot analysis of: TLR4 (**A**), MyD88 (**B**), IkB-α (**C**) and NF-kB (**D**) levels. For each analysis, *n* = 5 animals for each group were employed. The results were analyzed by one-way ANOVA, followed by a Bonferroni post hoc test for multiple comparisons. A *p*-value of less than 0.05 was considered significant. A *p*-value of less than 0.05 was considered significant. ^##^ *p* < 0.01 vs. sham, ** *p* < 0.01 vs. CCl4, *** *p* < 0.001 vs. CCl4.

In order to confirm SAG anti-inflammatory effects, Western blot analyses were conducted. TLR4 (Figure 6A) and MyD88 (Figure 6B) were significantly increased in CCl4-treated mice, as compared to the sham group. SAG administration significantly reduced their expressions. Next, the NF-kB pathway was examined. The sham group showed basal IkB-α cytosolic expression (Figure 6C) and poor nuclear NF-kB p-65 levels (Figure 6D). CCl4 intoxication importantly degraded cytosolic IkB-α and increased nuclear NF-kB. SAG administration restored IkB-α in cytosol (Figure 6C) and reduced NF-kB nuclear levels (Figure 6D).

### 2.7. Effects of SAG on Liver Fibrosis Induced by CCl4 Chronic Exposure 

**Figure 7:**
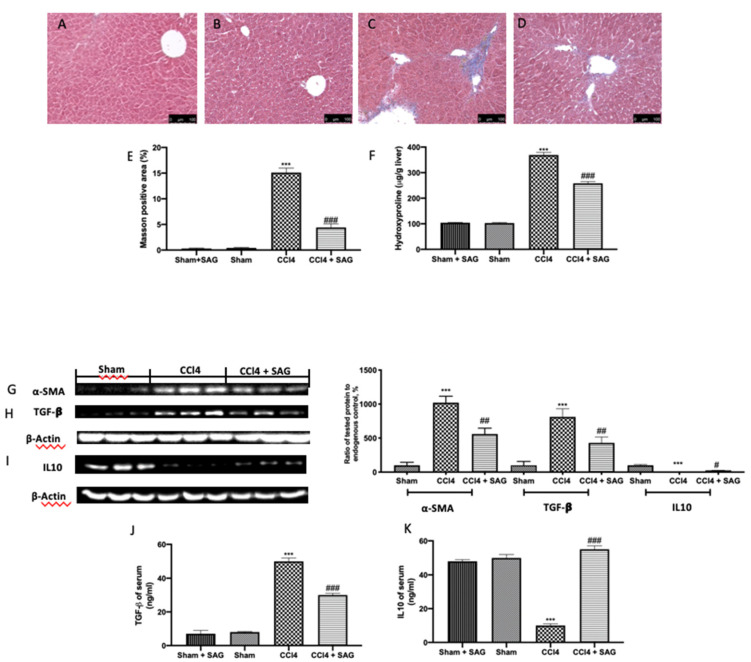
Administration of SAG reduced liver fibrosis induced by CCl4 injections: Masson trichrome staining: Sham + SAG (**A**), Sham (**B**), CCl4 (**C**), CCl4 + SAG (**D**), graphical quantification of Masson-positive area (**E**); Hydroxyproline content **(F**); Western blot analysis of: α-SMA (**G**), TGF-β (**H**) and IL10 (**I**) levels in the liver tissue; Serum levels of TGF-β (**J**) and IL10 (**K**). For each analysis, *n* = 5 animals for each group were employed. The results were analyzed by one-way ANOVA, followed by a Bonferroni post hoc test for multiple comparisons. A *p*-value of less than 0.05 was considered significant. A *p*-value of less than 0.05 was considered significant. ^#^ *p* < 0.05 vs. sham, ^##^ *p* < 0.01 vs. sham, ^###^ *p* < 0.001 vs. sham, *** *p* < 0.001 vs. CCl4.

To evaluate fibrosis, CCl4-induced Masson trichrome staining was performed. CCl4-treated animals showed an altered lobule structure through paraplastic connective tissue, and mild to serious fibrosis was detected (Figure 7C,E) compared to the sham group (Figure 7B,E). No differences were assessed between the sham and sham + SAG groups (Figure 7A,E). An assessment of hydroxyproline was used to test the collagen content in the liver tissue. The hydroxyproline content was also increased in CCl4-treated mice (Figure 7F), which was well in line with the upregulated levels of α-SMA (Figure 7G) and TGF-β (Figure 7H), whereas IL10 levels were decreased (Figure 7I) in the liver tissue. SAG administration significantly reduced the collagen deposition (Figure 7D,E), hydroxyproline content (Figure 7F) and α-SMA (Figure 7G) and TGF-β (Figure 7H) levels, whereas it increased IL10 expression (Figure 7I) in the liver tissue. Well in line with the tissue results, TGF-β levels (Figure 7J) increased in the serum with CCl4, whereas IL10 levels (Figure 7K) decreased. Both levels in the serum were normalized by SAG administration. 

### 2.8. Effects of SAG on Histopathological Alterations and Liver Function Induced by CCl4 Chronic Exposure

**Figure 8:**
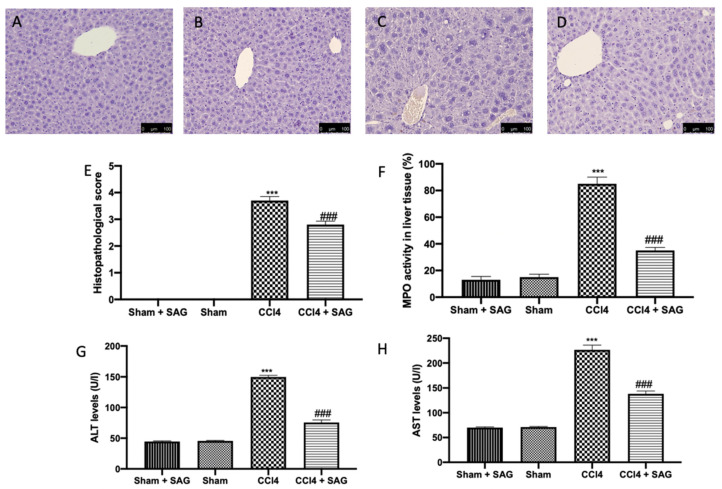
Administration of SAG reduced histological changes and liver dysfunction induced by CCl4 injections: Histological analysis: Sham + SAG (**A**), Sham (**B**), CCl4 (**C**), CCl4 + SAG (**D**), Histological score (**E**); MPO activity (**F**); ALT (**G**) and AST (**H**) levels. For each analysis, *n* = 5 animals for each group were employed. The results were analyzed by one-way ANOVA, followed by a Bonferroni post hoc test for multiple comparisons. A *p*-value of less than 0.05 was considered significant. A *p*-value of less than 0.05 was considered significant. ^###^ *p* < 0.001 vs. sham, *** *p* < 0.001 vs. CCl4.

In liver, CCl4 administration increased the myeloperoxidase (MPO) activity, which is used as an indicator of polymorphonuclear (PMN) cell infiltration (Figure 8F), liver cell damage and necrosis (Figure 8C,E), whereas the sham (Figure 8B,E) and sham + SAG (Figure 8A,E) groups showed normal histological architecture. CCl4 intoxication also compromised liver function, as shown by ALT (Figure 8G) and AST (Figure 8H) levels. SAG administration significantly ameliorated histological damage (Figure 8D,E), MPO activity (Figure 8F) and liver function (Figure 8G,H).

## 3. Discussion

Liver is an important organ involved in several activities, including bile acid secretion, the generation of blood clotting factors and detoxification. Liver injury may be induced by a variety of factors, including drugs, microbes, xenobiotics and several metabolites [19,20,21,22]. Previous studies have indicated that increasing GSH plasma levels has beneficial systemic effects [18], well in line with the described GSH depletion in non-alcoholic fatty liver disease patients compared with controls [23]. However, the oral administration of GSH does not significantly enhance GSH in plasma, while GSH derivatives have been described as able to cross the cell membranes and enhance the oral availability. SAG, a GSH precursor, is more stable in plasma, uptaken by cells and later converted to GSH. In this paper, we evaluated the hepatoprotective effects of SAG in a CCl4-model of liver injury, in which, the liver microsomal oxidizing systems related to cytochrome P-450 produce reactive metabolites of CCl4, including trichloroperoxyl radicals (CCl3O3·) or trichloromethyl radicals (CCl3). These reactive radicals induce lipid peroxidation, causing inflammation and hepatocellular damage and enhancing the production of fibrotic. The pre-treatment of primary hepatic cells with SAG followed by exposure to CCl4 protected cells against the cytotoxicity, indicating that the extracts counteracted the toxicity of products generated by the metabolism of CCl4. This result was corroborated by the analysis of ALT and AST liver enzymes, which overflow to the extracellular medium due to membrane permeability alterations after cellular injury. As expected, exposure to CCl4 induced a marked release of ALT and AST in primary hepatic cells, while the pre-treatment with SAG promoted the normalization of liver enzyme levels, indicating the protection of the cell membrane. SAG administration, thanks to its ability to maintain a cellular reductive state, enhanced antioxidants, including Nrf2, HO-1 and NQO-1, and restored SOD activity and GSH levels in the liver as well. SAG also reduced GSSG levels, lipid peroxidation and H_2_O_2_ liver levels. Accordingly, recent evidence focused the attention on the importance of the mitochondrial dysfunction induced by the sustained ROS in liver fibrosis [24]. Mitophagy is a useful mechanism that aims to remove impaired mitochondria, and is activated by organelle membrane depolarization [25]. Once activated, this mechanism leads to PINK1 stabilization on the mitochondrial outer membrane and to Parkin recruitment from cytosol. The increased oxidative stress characteristic on liver disfunction causes the impairment of mitophagy and accumulation of dysfunctional and damaged mitochondria. A failure of mitophagy or mitochondrial biogenesis affects hepatocellular function during ischemia/reperfusion [26] and cholestasis [27]. Moreover, interplay between these processes and oxidative metabolism, which may contribute to hepatic cell damage, has been suggested to occur in the steatotic liver [28,29]. 

Our results showed the protective effect of SAG administration in restoring mitophagy, as shown by the increased PINK1 and Parkin expressions in livers exposed to CCl4 intoxication. As already shown [30,31], CCl4 damage is closely associated with inflammation [32]. SAG administration strongly decreased pro-inflammatory cytokines TNF-α, IL-6, MCP-1 and IL-1β in both the serum and liver. This cytokine release is induced by several pathways, including the TLR4/MyD88, a well-known signaling involved in the CCl4-induced liver injury. In particular, TLR4 recruits the specific mediator MyD88, which triggers down-streaming signaling events for the NFkB phosphorylation and a consequent release of pro-inflammatory cytokines [33,34]. ROS interacts with NF-κB signaling pathways in many ways. The transcription of NF-κB-dependent genes influences the levels of ROS in the cell, and, in turn, the levels of NF-κB activity are also regulated by the levels of ROS [35]. NFkB is a transcription factor normally located in the cytoplasm and bound to the IKK complex. Thanks to the activation of the TLR4 pathway, Ikb-α is phosphorylated and degraded by proteasome, leading to NFkB release and translocation into the nucleus to increase the expression of targeting genes implicated in the inflammatory response [36,37]. Reducing inflammation and oxidative stress, SAG administration importantly reduced liver fibrosis, as confirmed by the decreased collagen deposition, assessed by Masson trichrome staining, and the reduced hydroxyproline contents and α-sma and TGF-β expressions, while it restored IL10 levels. Consequently, SAG administration attenuated histological liver damage, assessed by hematoxylin and eosin staining; MPO activity, used as an indicator of PMN cell infiltration [38,39,40]; and improved hepatic function, as shown by ALT and AST levels.

**Figure 9:**
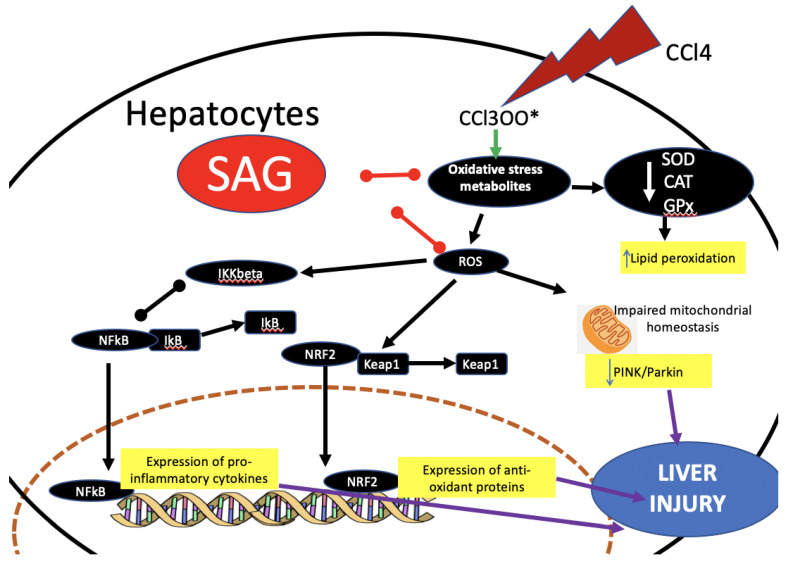
Graphical summary of SAG mechanism.

In conclusion, the present study indicated the potential protective effects of SAG against CCl4-induced liver damage. The hepatoprotective effects of SAG depend on its ability to reduce the generation of ROS, as well as pro-inflammatory signaling through the de-activation of TLR4/NF-κB signaling (Figure 9). Overall, the present study provides evidence for the protective effects of SAG against CCl4-induced liver injury and suggests SAG as a potential hepatoprotective agent used to prevent oxidative liver damage. 

## 4. Materials and Methods

### 4.1. Culture of Hepatocytes

Isolated hepatocytes were prepared from male C57BL/6 mice by the collagenase digestion [41]. Cells were purified by several centrifugations and cultured in DMEM medium supplemented with 10% FBS, HEPES (4.5 mM), penicillin (100 U/mL), L-glutamine (2 mM) and sodium bicarbonate (0.17 M). Cells were maintained under standard culture conditions at 37 °C and 5% CO_2_ in a humid environment and trypsinized for experiments whenever the cellular confluence was 70%.

### 4.2. Cytotoxicity Assay

The assessment of the hepatoprotective effect of SAG was performed using the 3-[4,5-dimethylthiazol-2-yl]-2,5-diphenyl tetrazolium bromide (MTT) reduction assay [42]. 

Cells (1 × 105 cells/well) were seeded in 96-well plates and maintained for 24 h in culture conditions in order to adhere to them. The experimental design involved pre-treatment of primary hepatic cells with SAG (at 0.25, 0.5, 0.75, 1.0, 1.25, 1.5 or 2.00 mM, dissolved in saline as stock solution and diluted in DMEM) for 1 h followed by exposure to CCl4 (4 mM diluted in 0.5% DMSO) for 6 h. Control group was carried out without CCl4 exposure. At the exposure time, the medium was exchanged and, after 21 h, 10 μL of MTT (5 mg/mL) was added to each well, and the plates were incubated for 3 h in culture conditions. Supernatants were removed and 100 μL of DMSO was added to each well to solubilize the formazan crystals. The absorbance was measured on a microplate reader at 570 nm.

### 4.3. Activities of ALT and AST in Cell Supernatants

An evaluation of the activities of the liver-specific enzymes alanine aminotransferase (ALT) and aspartate aminotransferase (AST) is recommended for the assessment of hepatocellular function [43]. For this purpose, primary hepatic cells (1 × 10^6^ cells/well) were seeded in 24-well plates, incubated for 24 h in culture conditions and subsequently pre-treated with SAG (at 2.00 mM) followed by exposure to CCl4. Afterwards, supernatants were collected and centrifuged at 5000 rpm for 5 min using a microtube centrifuge. Analyses of the enzymatic activities of AST and ALT were performed according to the manufacturer’s instructions (Sigma-Aldrich, Milan, Italy).

### 4.4. Animals

Sprague Dawley male rats (250 gr, Envigo, Milan, Italy) and C57BL/6 mice (male 20–22 g; age 6–8 weeks) were purchased from Envigo (Milan, Italy) and employed for this study. Messina University Review Board for the carefulness of animals permitted the research (211/2021-PR). All animal experiments agree with the new regulations in Italy (D.Lgs 2014/26), EU regulations (EU Directive 2010/63). 

### 4.5. Carrageenan-Induced Paw Edema (Preliminary Data)

Carrageenan-induced paw edema was performed as previously indicated by a subplantar injection of carrageenan (0.1 mL/rat of a 1% suspension in saline) (Sigma-Aldrich, Milan, Italy) into the right hind paw [44]. Increase in paw volume (mL) was measured using a plethysmometer (Ugo Basile, Varese, Italy) immediately prior to the carrageenan injection and every hour for 6 h (Supplemental Figure S1).

### 4.6. Experimental Groups

Respectively, mice were randomly assigned to different groups, as described below:

- Sham + SAG: mice were injected intraperitoneally with olive oil twice a week and were treated orally for 8 weeks with SAG (30 mg/kg) (Merk, CAS n 3054-47-5, AMBH95E07091) dissolved in saline.
- Sham: mice were injected intraperitoneally with olive oil twice a week.
- CCl4: mice were intraperitoneally injected with CCl4 1 mL/kg (diluted at 1:10 in olive oil) twice a week for 8 consecutive weeks to induce liver fibrosis [11].
- SAG: mice were administered with CCl4 to induce liver fibrosis as vehicle group and were treated orally for 8 weeks with SAG (30 mg/kg) dissolved in saline.

At the end of the experiment, blood samples and liver tissues were collected from the mice for further assays. The dose of SAG has been chosen based on experiments previously conducted in our laboratory. 

### 4.7. Analysis of Biochemical Indicators

Serum alanine aminotransferase (ALT) and aspartate aminotransferase (AST) levels were determined according to manufacturer’s instructions (Sigma-Aldrich). As for the hepatic hydroxyproline content, snap-frozen liver specimens were collected and the hydroxyproline content was quantified following the manufacturer’s instructions (Sigma-Aldrich) [11]. Determination of SOD activity was performed as already described [45]. SOD activity (U/μg protein) was determined using a microplate reader at 560 nm [46]. GSH levels were determined using a microplate reader at 412 nm and expressed as ng/mg wet tissue [45]. GSSG levels were determined using a microplate reader at 450 nm and expressed as ng/mg wet tissue (MyBiosource MBS749109). Glutathione peroxidase activity was estimated by measuring the oxidation of guaiacol in the liver of treated mice according to a standard method [47]. Lipoperoxidation was estimated using the thiobarbituric acid reactive substances (TBARS) test [48]. Briefly, liver tissue was weighed and homogenized in a 1.15% (*w*/*v*) KCl solution. A 100 mL aliquot of homogenate was then removed and added to a reaction mixture containing 200 mL 8.1% (*w*/*v*) lauryl sulfate, 1.5 mL 20% (*v*/*v*) acetic acid (pH 3.5), 1.5 mL 0.8% (*w*/*v*) thiobarbituric acid and 700 mL distilled water. Samples were then boiled for one hour at 95 °C and centrifuged at 3000× *g* for 10 min. The absorbance of the supernatant was measured spectrophotometrically at 532 nm. MDA levels were expressed as nmol/g wet tissue weight. Whole liver-derived lysates were diluted according to the manufacturer instruction (E-BC-K102-S) and incubated with ammonium molybdate reagent. H_2_O_2_ content can be calculated by measuring the absorbance value at 405 nm. Relative level of ROS was detected centrifuging liver tissue in the appropriate buffer (20 mmol/L Tir-HCl (pH 7.4), 20 mmol/L NaH_2_PO_4_, 5 mmol/L MgCl_2_, 130 mmol/L KCl and 30 mmol/L glucose) and incubating the supernatant with DCFH-DA (2′, 7′-dichlorodihydrofluorescein diacetate) for 15 min at 37 °C. Then, the reaction was terminated by adding 1 micromol/L of H_2_O_2_. The absorbance value was determined by fluorescence spectrophotometer [49].

### 4.8. Determination of Myeloperoxidase Activity

MPO activity in liver tissue was used as an indicator of polymorphonuclear (PMN) cell infiltration using a method previously described [50]. Briefly, tissue was weighed and homogenized in a solution containing 0.5% (*w*/*v*) hexadecyltrimethylammonium bromide dissolved in 10 mmol/L potassium phosphate buffer (pH 7.4) and centrifuged for 30 min at 20,000× *g* at 4 °C. An aliquot of supernatant was then removed and added to a reaction mixture containing 1.6 mmol/L tetramethylbenzidine and 0.1 mmol/L hydrogen peroxide (H_2_O_2_). The rate of change in absorbance was measured spectrophotometrically at 650 nm. 

### 4.9. ELISA

The levels of tumor necrosis factor (TNF)-α, interleukin (IL)-1β, IL-6 and monocyte chemoattractant protein (MCP)-1 in serum and liver tissue samples were determined by ELISA kits (R&D systems) [51], The levels of TGF-β and IL10 in serum were determined by ELISA kits (R&D systems) [52].

### 4.10. Histopathological Examination 

For histopathological investigations, liver tissues were fixed in formaldehyde solution (10% in PBS); histological sections were stained with hematoxylin and eosin (H&E) and evaluated using a Leica DM6 microscope (Leica Microsystems SpA, Milan, Italy) equipped with a motorized stage and associated with Leica LAS X Navigator software (Leica Microsystems SpA, Milan, Italy) [53]. Histopathologic scores were graded following the Ishak scoring system as follows: 0, no fibrosis; 1, fibrosis expansion of some portal areas ± short fibrous septa; 2, fibrosis expansion of portal areas ± short fibrous septa; 3, fibrosis expansion of most portal areas with occasional portal to portal bridging; 4, fibrosis expansion of portal areas with marked portal to portal bridging, as well as portal to central; 5, marked bridging with occasional nodules (incomplete cirrhosis); 6, cirrhosis, probable or definite [54,55,56]. Collagen deposition was evaluated by Masson trichrome staining performed according to the manufacturer’s protocol (Bio-Optica, Milan, Italy) [57]. 

### 4.11. Western Blot Analysis 

Western blot analyses were made as previously described [58]. Filters were blocked with 1× PBS, 5% (*w*/*v*) no-fat dried milk (PM) for 40 min at room temperature and then probed with one of the next primary antibodies: anti-TGF-β (Santa Cruz Biotechnology, sc-130348), anti-IL-10 (Santa Cruz Biotechnology, sc-8438) or anti-IkB-α (Santa Cruz Biotechnology, sc-1643), or anti- NF-kB p-65 (Santa Cruz Biotechnology, sc-8008), or anti-TLR4 (Santa Cruz Biotechnology, sc-293072), anti-MyD88 (Santa Cruz Biotechnology, sc-74532), or anti-α-SMA (Santa Cruz Biotechnology, sc-53015), or anti-Nrf-2 (Santa Cruz Biotechnology, sc-365949), or anti-HO-1 (Santa Cruz Biotechnology, sc-136960), or NQO-1 (Abcam), or anti-PINK1 (Santa Cruz Biotechnology, sc-517353), or anti-Parkin (Santa Cruz Biotechnology, sc-32282) in 1× PBS, 0.1% Tween-20, 5% *w*/*v* no-fat dried milk (PMT) at 4 °C overnight. Membranes were incubated with peroxidase-conjugated bovine anti-mouse IgG secondary antibody or peroxidase-conjugated goat anti-rabbit IgG (1:2000, Jackson ImmunoResearch, West Grove, PA, USA) [59]. Blots were also incubated with primary antibody against β-actin protein (1:10,000; Sigma-Aldrich Corp.) or lamin (1:10,000; Sigma-Aldrich Corp.), used as internal standards [60]. The relative expressions of the protein bands were detected and quantified by densitometry, as previously explained [61]. In the experiments including Western blot, a representative blot is displayed and densitometric analysis is related in each figure.

### 4.12. Statistical Evaluation

All values in the images and text are expressed as mean ± standard error of the mean (SEM) of N observations. For in vivo studies, N represents the number of animals. In experiments involving histology and immunohistochemistry, the illustrations represent the outcomes of at least three independent experiments. The results were analyzed by one-way ANOVA, followed by a Bonferroni post hoc test for multiple comparisons. A *p*-value of less than 0.05 was considered significant. A *p*-value of less than 0.05 was considered significant. # *p* < 0.05 vs. sham, * *p* < 0.05 vs. vehicle, ## *p* < 0.01 vs. sham, ** *p* < 0.01 vs. vehicle, ### *p* < 0.001 vs. sham, *** *p* < 0.001 vs. vehicle.

## Data Availability

The data used to support the findings of this study are available from the corresponding author upon request.

## Supplementary Materials

The following supporting information can be downloaded at: https://www.mdpi.com/article/10.3390/ijms23084429/s1.
