# Supplementary material for: S-Acetyl-Glutathione Attenuates Carbon Tetrachloride-Induced Liver Injury by Modulating Oxidative Imbalance and Inflammation
Source: Int J Mol Sci. 2022 Apr 17;23(8):4429. doi: 10.3390/ijms23084429 (PMC9024626; doi:10.3390/ijms23084429)
Supplement: Supplementary file 1 [file ijms-23-04429-s001.zip › ijms-1650929-supplemental.pdf]

Acute effects of SAG on carrageenan-induced paw edema: preliminary data

To investigate which dose of SAG could have an anti-inflammatory activity, we first studied the acute effects of SAG on a classical model of inflammation such as carrageenan-induced paw edema. To our best knowledge this derivate of GH has never been tested before even if other derivatives such as S-allyl-GSH have been already describe [19]. We tested three different doses 3, 10 and 30 mg/kg. Intraplantar carrageenan injection caused an important time-dependent increase in paw volume in carrageenan-injected rats until 6 h. SAG at doses of 3 and 10 mg/kg were not able to reduce, in a significant way, the paw edema, whereas the higher dose of 30 mg/kg was able to reduce, even if statistically significant only at 6 h post carrageenan, the paw inflammation (Figure S1).

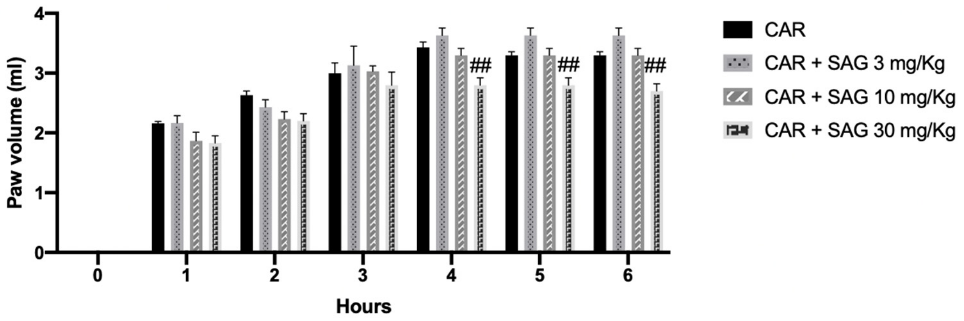

Figure S1. Preliminary data of acute effects of SAG at different doses on carrageenan-induced paw edema: Paw volume. For this analysis n = 5 animals for each group were employed. The results were analyzed by two-way ANOVA, followed by a Bonferroni post-hoc test for multiple comparisons. A p-value of less than 0.05 was considered significant. A p-value of less than 0.05 was considered significant. ## $p < 0.01$ vs. vehicle.
